# Fetal Brain Abnormality Classification from MRI Images of Different Gestational Age

**DOI:** 10.3390/brainsci9090231

**Published:** 2019-09-12

**Authors:** Omneya Attallah, Maha A. Sharkas, Heba Gadelkarim

**Affiliations:** 1 Department of Electronics and Communications, College of Engineering and Technology, Arab Academy for Science and Technology and Maritime Transport, Alexandria, P.O. Box 1029, Egypt; msharkas@aast.edu (M.A.S.); heba.mehery@gmail.com (H.G.); 2 Department of Computer and Communication Engineering (SSP), Faculty of Engineering, Alexandria University, Alexandria 21526, Egypt

**Keywords:** biomedical image processing, ensemble classification, fetal brain abnormalities, principal component analysis (PCA), feature extraction

## Abstract

Magnetic resonance imaging (MRI) is a common imaging technique used extensively to study human brain activities. Recently, it has been used for scanning the fetal brain. Amongst 1000 pregnant women, 3 of them have fetuses with brain abnormality. Hence, the primary detection and classification are important. Machine learning techniques have a large potential in aiding the early detection of these abnormalities, which correspondingly could enhance the diagnosis process and follow up plans. Most research focused on the classification of abnormal brains in a primary age has been for newborns and premature infants, with fewer studies focusing on images for fetuses. These studies associated fetal scans to scans after birth for the detection and classification of brain defects early in the neonatal age. This type of brain abnormality is named small for gestational age (SGA). This article proposes a novel framework for the classification of fetal brains at an early age (before the fetus is born). As far as we could know, this is the first study to classify brain abnormalities of fetuses of widespread gestational ages (GAs). The study incorporates several machine learning classifiers, such as diagonal quadratic discriminates analysis (DQDA), K-nearest neighbour (K-NN), random forest, naïve Bayes, and radial basis function (RBF) neural network classifiers. Moreover, several bagging and Adaboosting ensembles models have been constructed using random forest, naïve Bayes, and RBF network classifiers. The performances of these ensembles have been compared with their individual models. Our results show that our novel approach can successfully identify and classify numerous types of defects within MRI images of the fetal brain of various GAs. Using the KNN classifier, we were able to achieve the highest classification accuracy and area under receiving operating characteristics of 95.6% and 99% respectively. In addition, ensemble classifiers improved the results of their respective individual models.

## 1. Introduction

In previous years, magnetic resonance imaging (MRI) has been efficiently used to study the functionality of the human brain. MRI images are capable of establishing and accomplishing unmatched medical examination to determine whether the brain has any abnormalities. Lately, in addition to scanning adult brains, this imaging technique has also been used as a new non-invasive instrument designed for improving and checking fetal brain development in the uterus. Since around 3 in every 1000 pregnant women carry fetuses with various types of brain abnormalities, fetal brain monitoring is important. Moreover, a series of neuropathological variations can occur, several of which are connected to serious clinical morbidities [1]. A MRI scan for a fetus provides great details of the soft tissue and the structure of the brain. Therefore, it could be applied for primary identification of brain abnormalities and tumors with no need for medical interferences [2,3]. Identifying these defects in a primary phase using machine learning techniques has several advantages. First, it helps doctors to confer an accurate diagnosis, allowing doctors to provide parents with an understanding of the disease in order to prepare them for dealing with the abnormality. Second, it helps in managing the pregnancy and problems that might occur during this period. Third, it improves the quality of diagnosis and aids the decision for the best suitable treatment plan [4].

Machine learning techniques make use of the ability of computers to train and learn without being explicitly programmed. Machine learning can be used for medical images to assist doctors with making accurate medical diagnoses. Additionally, it can reduce diagnostic errors by clinicians. Moreover, machine learning reduces the time scale and effort made during an examination [5]. Recently, machine learning techniques have been used on fetal brain MRI images for the early detection of abnormalities, as well as for the identification and classification of these abnormalities [6]. Most of the previous work that used fetal brain images focused on segmenting fetal brain images to detect abnormalities or separating the fetal brain from the rest of the body. Fewer studies considered the use of machine learning methods to identify defects existing in the fetal brains [7,8]. To the best of our knowledge, previous studies that applied classification methods to fetal brain MRI images are limited to References [9,10,11]. Although the first two studies performed classification on fetal MRI brain images, the authors only classified a brain defect in newborns called small for gestational age (SGA). They first assumed that textural patterns of fetal brain MRI images are associated with newborn neurobehavior. Then, they constructed a support vector machine (SVM) classifier for predicting the SGA abnormality after the birth of the fetus (becoming a neonate). These methods have several limitations. First, these approaches only used fetal images of 37 GAs, in contrast to applying a wide range of fetal GAs. Second, their methods only classify one type of abnormality; namely, SGA for newborns. Third, mapping the fetal MRI to its corresponding MRI after birth is an essential hypothesis needed to classify the SGA abnormality. Additionally, the authors used two small datasets which only have 91 and 83 images, respectively. Lastly, the brain of the fetus was clipped in a manual manner, which is a time-wasting process. The authors of Reference [11] classified several fetus abnormalities, but the highest classification accuracy achieved was limited to 80%.

The literature shows that the majority of work done on detecting and classifying brain abnormalities in early age was limited to infants, neonates, and preterm infants, rather than fetuses. Ball et al. [12] used the independent component analysis (ICA) technique with a SVM classifier to identify brain defects in preterm infants. Symser et al. [13] used support vector regression (SVR) to predict the GA of infants. He et al. [14] proposed a stacked spare auto-encoder (SSAE) framework based on an artificial neural network (ANN) classifier to predict preterm defects. They also used an SVM classifier to detect autism. Similar work was done by Jin et al. [15] to identify infants with autism.

The key objective of this article is classifying between normal (healthy) and abnormal (unhealthy) brains. As it is clear from the previous two paragraphs, the work done in fetal brain classification is very limited. Most of the previous related research dealing with brain defect classification in a primary age have been for infants and preterm neonates. As stated before, the early detection of brain abnormalities is important as it helps in managing the pregnancy and problems that might occur during this period. It could also enhance the diagnosis process and follow up plans. Therefore, this article proposes a novel approach to identify defects in fetal brain scans. This approach is divided into five phases: Segmentation, enhancement, feature extraction, feature reduction, and classification. First is the segmentation, where the brain of the fetus is defined as the region of interest (ROI). It is cropped semi-automatically from other fetal parts. This is performed in three steps. In the first step, the cerebrospinal fluid (CSF), maternal tissues, and strips of the skull are removed from the fetal brain MRI using morphological operations. In the next step, area-based segmentation (ABS) is performed, where the largest area in the image is detected and segmented using region properties and watershed transform methods. In the last step of the segmentation phase, a minor ROI containing the fetal brain abnormality (FBA) is delimited and clipped out of the fetal scan semi-automatically. The following phase is called the enhancement phase. In this phase the minor ROI image is improved using a mixture of filters to increase its contrast. Subsequently comes the feature extraction phase, where six subsets of features are extracted using different textural analysis feature extraction methods. Two of these feature subsets have a high dimensional space; therefore, they are reduced using the principal component analysis (PCA) technique in the feature reduction step. The last phase is the classification phase, where the six subsets of features are used to train and test different classification models, such as random forest decision tree, naïve Bayes, radial basis function (RBF) network, diagonal quadratic discriminates (DQDA), and K-nearest neighbour (K-NN). Several bagging and Adaboosting ensembles are also constructed using naïve Bayes, the RBF network, and random forest classifiers to improve the prediction of their respective individual classifiers.

**Figure 1:**
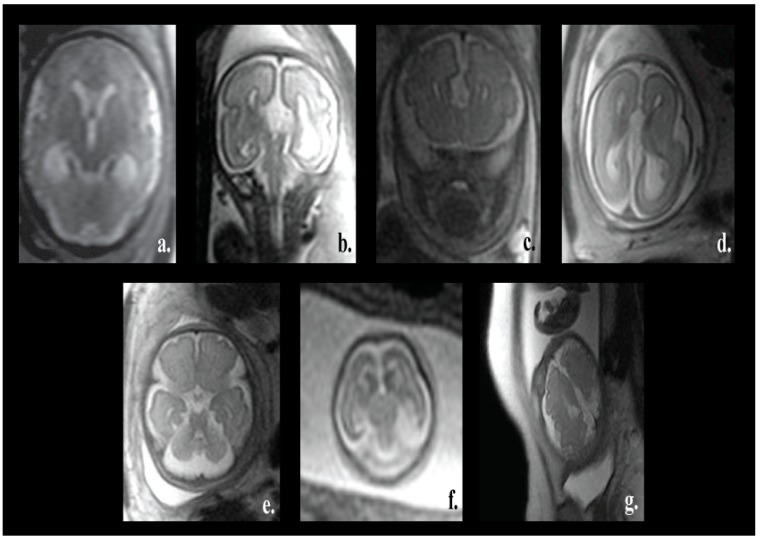
Different fetal brain abnormalities: (**a**) Agenesis of the septi pellucidi, (**b**) Dandy-Walker malformation, (**c**) colpocephaly, (**d**) agenesis of the corpus callosum, (**e**) mega-cisterna manga, (**f**) cerebellar hypoplasia, and (**g**) polymicrogyria.

One of the major advantages of our proposed pipeline is its ability to differentiate between normal and various fetal brain abnormalities, including agenesis of the septi pellucidi, Dandy–Walker variant/malformation, colpocephaly, agenesis of the corpus callosum, mega-cisterna magna, cerebellar hypoplasia, and polymicrogyria. All of these abnormalities are shown in Figure 1.

The corpus callosum is the curved structure that connects the left and right hemispheres of the brain. Agenesis of the corpus callosum (shown in Figure 1d) is a brain disorder in which the corpus callosum is partially or completely absent [16]. The septi pellucidi is a tinny tissue formed in the midline of the fetal brain. Agenesis of the septi pellucidi (shown in Figure 1a) is a fetal brain defect that affects the development of the septi pellucidi. It commonly occurs in association with other brain disorders [17,18]. Dandy–Walker syndrome (shown in Figure 1b) is a congenital malformation that affects the cerebellum, the third, and the fourth ventricles of the brain. It is characterized by partial or complete agenesis of the cerebellar vermis, cystic dilatation of the fourth ventricle, and the enlargement of the posterior fossa [19,20]. Colpocephaly (shown in Figure 1c) is a fetal brain abnormality in which the posterior of the lateral ventricles of the fetal brain are ballooned. It is characterized by an impaired intellect and microcephaly (an abnormally small head) [21]. Mega-cisterna magna (Figure 1e) is another brain defect which is characterized by the enlargement of the cisterna, cerebellar hemispheres, and morphologically intact vermis. The expansion of the cisterna magna measures more than 10 mm from the posterior aspect of the vermis to the internal aspect of the skull arch [22]. Cerebellar hypoplasia (shown in Figure 1f) is one more neurological disorder in which the cerebellum is partially developed and its size is small. Polymicrogyria (shown in Figure 1g) is a complex congenital malformation where the surface of the fetal brain normally has many ridges or folds, called gyri [23]. Friede et al. [24] defined polymicrogyria as “an abnormally thick cortex formed by the piling upon each other of many small gyri with a fused surface”.

Another advantage of the proposed approach is that the segmentation phase is a semiautomatic technique, saving the time and effort of manual segmentation. The proposed method was constructed and tested on a dataset consisting of 227 images, which is much larger when compared to the 91 and 45 images that have been used in previous studies [9,10]. Most importantly, the proposed approach can be applied for a wider range of fetal GAs and is not limited to a specific age.

The remainder of this paper is organized as follows: Section 2 introduces the methods and materials. This is followed by Section 3, which describes the results and comparative analysis of the proposed method. Section 4 confers the performance of our method and, finally, Section 5 concludes this article.

## 2. Materials and Methods

### 2.1. Data Acquisition

The dataset used in this paper is a public dataset available online [25]. It was collected by a medical team at Harvard medical school. At the institution where the clinical indication, such as evaluations of adnexal mass, studies were performed, pregnant patients signed an informed consent regarding MRI exposure. In addition, institutional review board approval was obtained and informed agreement was obtained from the patients. Therefore, our study did not seek the ethics committee/IRB to approve our study.

The dataset comprises 227 MRI scans for fetuses, with the GA extending between 16–39 weeks. More description is available in Reference [25]. Piles of T2 weighted fetal scans in coronal, pivotal, and sagittal planes were acquired with a solitary shot, half-Fourier, RARE grouping strategy. As a consequence of the fetal movement during the checkup, every obtaining fills in as the scout for the consequent procurement. A typical arrangement utilizes a reverberation dividing of 4.2 msec, an echo time T_E_ of 60 msec, a reverberation train of length equivalent to 72, 1 obtaining of 4 mm segment thickness, a 24 × 24 cm field of view, and 192 × 256 procurement grids. A 130-degree refocusing heartbeat was utilized to limit the amount of the radio-recurrence (RF) control statement. The procurement time per shot was only 430 msec for each cut.

### 2.2. The Proposed Method

**Figure 2:**
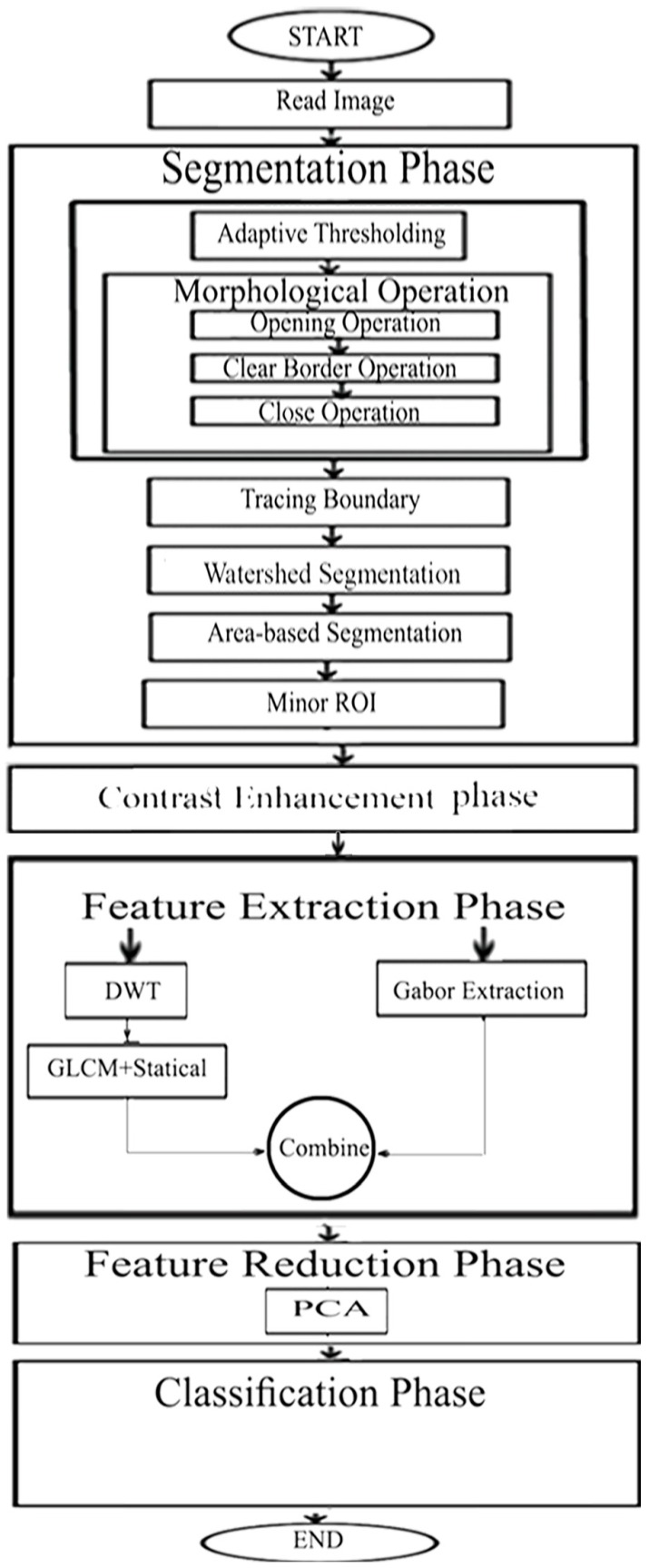
A diagram describing different phases of our novel approach.

The proposed framework is presented in five phases. The first was to segment the fetal brain region and then to enhance this region. Afterwards, came extracting significant features from this region. Next was reducing the dimension of the feature space. The final phase was classifying this region to identify whatever was normal or abnormal. A diagram describing the steps of our algorithm is presented in Figure 2. In the first phase, the brains of the fetuses, representing the ROI, were segmented out from both the maternal tissues and the rest of the fetal body using morphological operations, adaptive thresholding, watershed segmentation, and region properties techniques. Then, the part of the brain where the defect occurred was delineated and cropped. This part presents the minor ROI. Afterwards, an enhancement process was made on this part to increase its contrast using local and global filters. Later, several feature extraction methods, including Gabor filters, discrete wavelet transforms (DWT), and gray-level co-occurrence matrix (GLCM) were used to extract six subsets of features. These feature subsets are described in more detail in the feature extraction subsection. Two of the six subsets of features had a high feature space. Therefore, the principal component analysis (PCA) technique was used to reduce their feature space dimension. Finally, the six subsets of features were used to train and test several classification models. The following subsections provide a detailed description of the algorithm.

#### 2.2.1. Segmentation Phase

The segmentation phase consists of six steps. These steps are as follows: Adaptive thresholding, morphological operations, tracing boundary, watershed segmentation, area-based segmentation, and minor ROI extraction steps. These phases are discussed in detail in this section.

**Adaptive Thresholding Step**: Initially, the CSF and the amniotic fluid that surround the skull of the fetal brain are removed using an optimum onset ***T***. An adapting local thresholding technique [26] is employed to distinguish the pixels of an image into two classes, i.e., forefront and background. An image ***I*** is created from the input scan ***I*** once this step is done, using Equation (1).

(1) $$I_{binary}(i,j)=\left\{ \begin{array}{ll} 1, & if\;I(i,j)\geq T\;\\ 0, & \mathrm{otherwise} \end{array}\right.,$$

**Morphological Operation Step**: In this step, several morphological operations are used to convolve the binary image with the structuring element (disk, line, sphere, etc.) [27]. In the proposed framework three morphological operations are applied; namely, opening, clearing border, and closing operations, respectively.

The maternal areas associated with the outer part of the fetal skull are eliminated from the ***I_binary_*** image using a morphological opening operation using Equation (2). The image produced is called the opened image ***I_open_***.

*I_open_* = (*I_binary_* ⊖ *SE*) ⊕ *SE*, (2)

where ⊖ and ⊕ are the erosion and dilation operators, respectively, and *SE* stands for a structuring element representing a disk shape of radius 1.

The clear border operation is used to separate the outer part that is lighter than the surroundings, connecting the image border with the ***I_open_*** image. This operation is performed in order to obtain the ***I_clear_*** image, which is the image excluding the maternal tissue.

The closing operation is used to fill in the gaps within the skull and to smoothen the outer edges, which were damaged after the open operation, using Equation (3). The image produced after the closing operation is called ***I_close_***. Note that to protect the image from deformation, the ***SE*** in the closing operation must be the same value as the ***SE*** in the opening operation.

*I_close_* = (*I_clear_* ⊕ *SE*) ⊖ *SE*, (3)

**Tracing Boundary Step**: In this phase, the limits of the image ***I_close_*** are arranged into parent and children limits. The parent limits are the external limits of the skull. The children limits are the openings inside the skull, which are encompassed by the parent limits. Afterwards, these limits are ranked sorted descendingly to form a binary image ***I_region_*** describing parents and children.

**Watershed Segmentation Step:** A watershed is a ridge that separates areas drained by different water or river systems. The watershed segmentation technique is based on the same concept. Segmentation by the watershed method is a local approach. It utilizes a watershed transformation technique to decide the “watershed edge lines” or “catchment bowls” from a binary image by thinking about it as a surface, where the dark pixels are viewed as the low height and the white pixels are the high rise [28].

This method is applied to the ***I_region_*** image to eliminate the links between parental parts and the fetal skull. This is done in several steps. Initially, the distance transform ***d*** between the two white connection areas (fetal brain and outer part) is calculated. Then, the watershed transform is applied to the image. Afterwards, the extended-minima transform [27] is applied to compute the regional minima (minimum region) of the H-minima transform. The regional minima are the regions of connected pixels in an image with a constant intensity value. These regions are surrounded by border pixels having a high *d* value. Next, the distance transform ***d_new_*** is calculated again using the impose minima method. Impose minima is an algorithm used to modify the gray-scale image by using morphological reconstruction operations. The ***d_new_*** is then used to replace ***d***. This step is applied to avoid the over-segmentation problem with the watershed transform before the watershed transform is re-applied. The image following watershed segmentation is called ***I_watershed_***.

**Figure 3:**
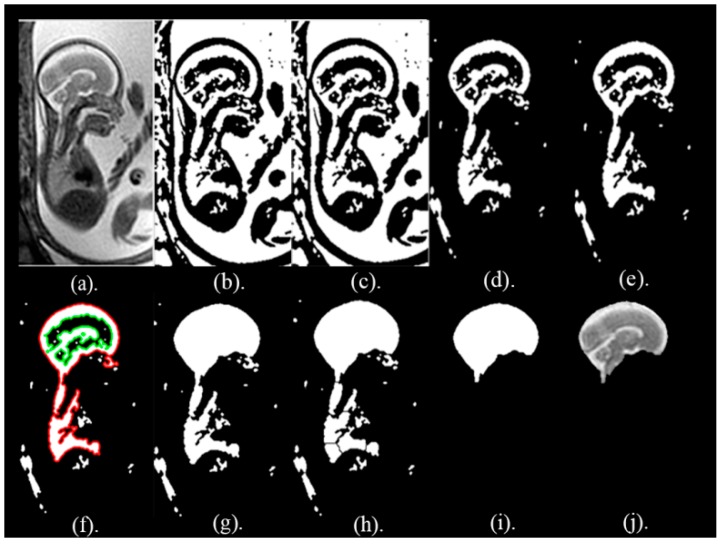
Fetal brain pictures the different steps of the segmentation phase. (**a**) The original fetal brain picture *I*. (**b**) Binary picture when adaptive thresholding was employed, *I_binary_*. (**c**) Binary picture when opening morphological operation was used, *I_open_*. (**d**) Binary picture *I_clear_* after using clear border process. (**e**) Binary picture when the closing process was made. (**f**) Binary picture presenting the parent boundaries in red and the children in green. (**g**) Binary picture applying the tracing boundary process, *I_region_*. (**h**) Binary picture when using watershed approach, *I_watershed_.* (**i**) Binary picture when applying the ABS process *I_ABS_*. (**j**) Picture of the fetal brain after the segmentation process, *I_final_*.

**Area Based Segmentation (ABS) Step:** In this step, the region property method is used to calculate the area of the objects formed in the ***I_watershed_*** image. These areas are arranged descendingly and the object with the biggest area is taken out to a create the segmented picture ***I_ABS_***. The ***I_ABS_*** image is used as a mask to extract the whole fetal brain image, ***I_final_***, from the original picture, ***I***, using the formula expressed in Equation (4). The pictures following these segmentation steps are shown in Figure 3.

(4) $$I_{final}(i,j)\;=\;\left\{ \begin{array}{ll} I(i,j), & \;if\;I(i,j)=1\;\\ 0, & Otherwise \end{array}\right.,$$

**Figure 4:**
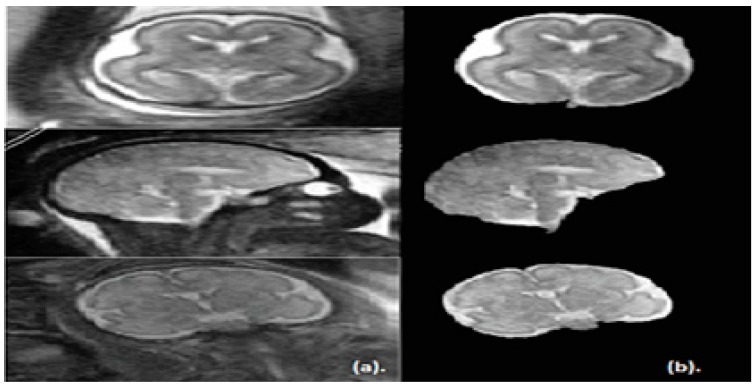
The fetal brain images in the sagittal, coronal, and axial planes. (**a**) Before segmentation and (**b**) after the proposed segmentation step.

Note that, the segmentation step of the proposed approach is applied on the sagittal, coronal, and axial planes of brain images. The segmented images for the three planes are shown in Figure 4.

**Figure 5:**
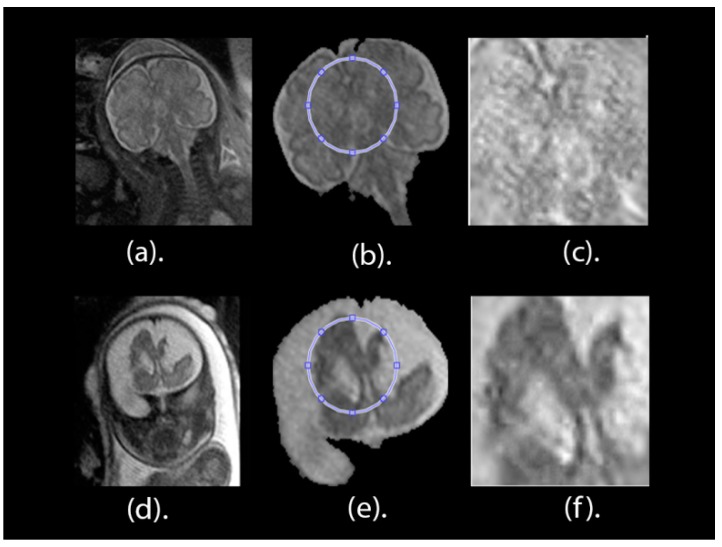
Brain pictures after contrast improvement and taking out the minor ROI. (**a**) is the full healthy brain picture afore segmenting the image. (**b**) The outlined ROI of the healthy fragmented brain. (**c**) The fragmented and improved ROI for a healthy brain. (**d**) The full unhealthy brain picture before segmenting the image. (**e**) The outlined ROI of the unhealthy fragmented brain. (**f**) The fragmented and improved ROI for an unhealthy brain.

After the whole fetal brain is segmented and detected as ***I_final_***, a minor ROI image is delineated for all fetal scans. This process depends on both the alterations of the grey levels of the image and the structure and shape of the brain. The upper right part of the scan, representing a minor ROI borderline, is selected. Afterwards, this region is separated out of the remaining parts of the brain image to form an image named as ***I_final_***. Figure 5a,d present the full brain scans afore the segmentation process for healthy and unhealthy classes, correspondingly. Figure 5b,e present the pictures of the brain ***I_final_*** after the segmentation procedure for healthy and unhealthy classes, correspondingly. The minor ROI for a healthy brain is displayed in Figure 5c and the minor ROI for an unhealthy brain is displayed in Figure 5f.

#### 2.2.2. Contrast Enhancement Phase

In this step, the minor ROI image is enhanced using a combination of local and global stretching contrast enhancement methods [29]. The global stretching contrast enhancement method is used to improve the low contrast for the image. This global enhancement algorithm avoids noise formation and the other ringing artifacts in the medical image. In the global contrast enhancement method, when a high contrast occurs, this results in the underexposure of some partitions of the image and the overexposure on other partitions. The local stretching contrast image enhancement algorithm increases the noise for the image when high contrast occurs.

This process is made to improve the contrast and reserve the intensity in the brain part. Additionally, this process increases the classification accuracy of our new presented approach. The fragmented minor ROI after contrast improvement for a healthy cerebrum is displayed in Figure 5c and the fragmented minor ROI after contrast improvement for an unhealthy cerebrum is shown in Figure 5f.

#### 2.2.3. Feature Extraction phase

Texture analysis feature extraction methods are commonly used for extracting features from MRI images [30,31,32,33]. These feature extraction methods are based on the textural characteristics of the image. There are many methods for extracting textural features, including the discrete wavelet transform (DWT), the Gabor filter, and the gray-level co-occurrence matrix (GLCM). These methods usually yield reasonable classification accuracy, especially when they are combined together [33,34]. For this reason, they were used in this study to extract several subsets of features. These feature extraction methods are discussed in this section.

##### Gabor Filters

A Gabor filter is a popular filter which is used to extract textural descriptors by investigating the frequency domain of the image [35]. It uses a Gaussian kernel function modulated with a complex sinusoidal plane wave. Gabor filters have the ability to perform multi-resolution decomposition, which produces a different frequency and orientation representation, similar to that of the visual system of human beings. Gabor filters demonstrate the fine dissimilarities between different textures in an image by analyzing the image to determine whether it has any specific frequency content with a specific orientation located in a small area around the region of analysis [36]. They are performed by multiplying the Gaussian function centered at different frequencies with the Fourier transform (FT) of the image and then applying the inverse fast Fourier transform (IFFT). It is important to choose the central frequency of each Gaussian function to ensure that all the frequencies of the image are covered [37]. Gabor filters consist of complex exponentials centered at a certain frequency and multiplied by a Gaussian envelop. Features are extracted with the Gabor filter using Equation (5).

(5) $$f(x,y)=e^{\frac{-{\left(x-x_{p}\right)}^{2}}{2\sigma_{x}^{2}}}-e^{\frac{-{\left(y-y_{p}\right)}^{2}}{2\sigma_{y}^{2}}}*e^{-i(w_{xo}x+w_{yo}y)},$$

where *x* and *y* are the positions of the pixels in an image, $\sigma_{x},\;and\;\sigma_{y}$ are the standard deviation (std.) of the Gaussian function belonging to *x* and *y* directions, and $w_{xo},{\;and\;w}_{yo}$ are the central frequencies of the *x* and *y* directions.

In this paper, an array of Gabor filters of different frequencies and orientations were used to localize different subsets of frequency and orientation features in the input image. Orientation angles are between (0, 150) in steps of 30 degrees. The wavelength ranges from 42 up to the hypotenuse length of the input image. Several Gabor magnitudes are extracted which are then passed through a simple Gaussian low-pass filter to smooth the Gabor magnitude information.

##### Discrete Wavelet Transform (DWT)

Discrete wavelet transform (DWT) is the most commonly used algorithm to extract features in the medical image processing field [33,38]. DWT is used to analyze signals and images. DWT provides time-frequency representation by decomposing a signal or an image using a set of orthogonal basis functions (Ortho-normal). DWT has a set of transforms each with a different set of the wavelet basis functions. For a signal to be analyzed, a 1-D DWT is used to decompose the input signal ***S***. This is done through a convolution process using a low pass and a high-pass filter. Afterwards, a dyadic decimation is made. The dyadic decimation is a down-sampling operation to overcome the aliasing problem. After the 1-D DWT is applied to ***S***, two sets of coefficients will be generated. These two sets are the approximation coefficients CA_1_ and detail coefficients CD_1_.

For 2-D images, a one level 2-D DWT, where a 1D-DWT is applied for each dimension separately to obtain four sets of coefficients, is used. The four components produced after the 2-D DWT are the approximation coefficients, CA_1_, and three detail coefficients, CD_1_. These detail coefficients include the horizontal, vertical, and diagonal coefficients, respectively. The 2-D DWT may be also applied to images to perform multiple decomposing stages. This is made through convolving the approximation components produced in the previous decomposition level into numerous high and low-pass filters [39,40].

In the proposed method, decomposing stages of DWT were made to the images. The four decomposition levels of 2D-DWT improve the performance when used with a brain MRI, as mentioned by Reference [41]. A Meyer wavelet (dmey) with mode periodization is employed as a wavelet basis function. After performing four level decompositions, the horizontal, approximate, diagonal n, and vertical coefficients of the 4th decomposing stage were extracted to form a feature subset.

##### Grey-Level Co-occurrence Matrix (GLCM)

The GLCM algorithm is a popular method for extracting texture features from images [42]. It is applied to mine surface features, such as correlation, homogeneity, contrast, correlation, and energy, in the images. The features extracted are calculated using Equations (6)–(9).

(6) $$Contrast=\overset{G-1}{\underset{n=0}{\sum }}n^{2}\left\{\overset{G}{\underset{i}{\sum }}\overset{G}{\underset{J}{\sum }}P\left(i,j\right)\right\}\;,\;\left|i-j\right|=n\;,$$

(7) $$Correlation=\;\overset{G}{\underset{i}{\sum }}\overset{G}{\underset{j}{\sum }}\frac{\left\{i*j\right\}*P\left(i,j\right)-\left\{\mu_{x}*\mu_{y}\right\}}{\sigma_{x}*\sigma_{y}\;},\;\;$$

(8) $$Energy=\;\sum_{i}^{G-1}\sum_{j}^{G-1}{\left(P\left(i,j\right)\right)}^{2},$$

(9) $$Homogeneity=\;\overset{G-1}{\underset{i}{\sum }}\overset{G-1}{\underset{j}{\sum }}\frac{P\left(i,j\right)}{1+\left|i-j\right|},$$

where *P* (*i*, *j*) is a marginal joint probability gray level co-occurrence matrix.

##### Statistical Descriptive Features

Statistical descriptive feature extraction is a method which extracts statistical features from a signal or picture. Those statistical variables comprise the entropy, variance, root mean square (RMS), mean, standard deviation (std), skewness, smoothness, kurtosis, and inverse difference moment (IDM) [43]. These features are mined from the minor ROI image using Equations (10)–(18).

(10) $$Mean\;\left(µ\right)=\frac{1}{N}\overset{N}{\underset{i=1}{\sum }}A_{i},$$

(11) $$Variance\left(A_{1}\ldots \ldots \;A_{N}\right)=\frac{1}{N-1}\overset{N}{\underset{j=1}{\sum }}{\left(A_{j}-\mu \right)}^{2},$$

(12) $$Std\left(\sigma \right)=\sqrt{Variance\left(A_{1}\ldots \ldots \;A_{N}\right)},$$

(13) $$Skewness\left(A_{1}\ldots \ldots \;A_{N}\right)=\;\frac{1}{N}\overset{N}{\underset{j=1}{\sum }}{\left[\frac{A_{j}-\mu }{\sigma }\right]}^{3},$$

(14) $$Entropy=\;\overset{G}{\underset{i}{\sum }}\overset{G}{\underset{j}{\sum }}P\left(i,j\right)*\;\log\left(P\left(i,j\right)\right),\;$$

(15) $$IDM=\;\overset{G}{\underset{i}{\sum }}\overset{G}{\underset{j}{\sum }}\frac{1}{1+{\left(i,j\right)}^{2}}P\left(i,j\right),$$

(16) $$Kurtoisis\left(A_{1}\ldots \ldots \;A_{N}\right)=\;\{\frac{1}{N}\overset{N}{\underset{j=1}{\sum }}{\left[\frac{A_{j}-\mu }{\sigma }\right]}^{4}\}-3,\;\;$$

(17) $$RMS=\overset{G}{\underset{i}{\sum }}\overset{G}{\underset{j}{\sum }}P\left(i,j\right)\left(\frac{\sqrt{\sum_{i,j=1}^{MN}{\left|{µ}_{i,j}\right|}^{2}}}{G^{2}}\;\right),$$

(18) $$Smoothness=1-\frac{1}{1+\sum_{i}^{G}\sum_{j}^{G}P\left(i,j\right)},$$

In the feature extraction phase, textural features extracted from the minor ROI are categorized into six subsets of features. The first subset represents features extracted using DWT (196 features). The second subset represents those features extracted using the Gabor filter (183,618 features). The third subset of features is those extracted using the gray-level co-occurrence matrix (GLCM) and statistical features extracted from the minor ROI in the spatial domain (13 features). The fourth subset represents features combined from the DWT, GLCM, and statistical variables describing the minor ROI in the spatial domain (209 features). The fifth subset represents GLCM and statistical features extracted from DWT coefficients (13 features). The sixth subset (183,631 features) represents features extracted from the Gabor filter feature extraction method combined with GLCM and statistical descriptive features extracted from DWT coefficients (fifth subset).

As noted, the DWT is used for feature extraction in two ways. The first one is the combination of the approximation, horizontal, vertical, and detail coefficients to form the feature vector with size A. The second method is the extraction of statistical features from the previously generated feature vector to form a new one with size B.

Some of these feature subsets, such as feature subset two and six, had high dimensional space (183,618 and 183,631 features). For this reason, a feature reduction algorithm was needed in the next step to reduce the feature dimension of these subsets (two and six). In the next section, the feature reduction process used in this paper is discussed.

#### 2.2.4. Feature Reduction Phase with Principal Component Analysis

Principal Component Analysis (PCA) is a common dimension-reduction technique [44]. This technique is used to compress the size of a large set of features to a new small set of features by transforming the correlated features into a set of uncorrelated features. This transformation is done by projecting the data onto a new orthogonal basis to present a new set of features with high variance. This new set consists of the principal components, which are formed through linear combinations of the initial features. These principal components are orthogonal to each other, so there are no redundant features. The first principle components contain much of the variance that exists in the original data. In the proposed method, the PCA was applied to feature subsets two and six, extracted in the previous step.

#### 2.2.5. Classification Phase

In this step, the feature sets produced during the extraction and reduction processes are employed to create and validate several classifiers models. Those models consist of diagonal quadratic discriminates analysis (DQDA), an RBF neural network, k-nearest neighbor (K-NN), a random forest decision tree, and naïve Bayes classifiers. Moreover, several bagging and Adaboosting ensemble models were constructed using naïve Bayes, random forest decision tree, and RBF neural network classifiers to determine whether they improve the performance of their individual models. For validating the performance of these models, the five-fold cross validation method was adapted.

##### K-nearest neighbor (K-NN)

This is a straightforward classification method. K-NN is a conventional non-parametric classifier. Despite its simplicity, K-NN has the power to accomplish high classification results in medical applications [45,46]. It allocates a class value to each data point in the training set by investigating the class values of its K nearest neighbors [47]. K-NN calculates the instance that needs classification from other instances in the training set of data features to assign a class value. The metric employed in this article to measure the distance was the cosine metric. The number of the nearest neighbors used during the prediction method for classifying each point was 10 and the inverse distance was chosen to be the distance weighing function [48].

##### Quadratic Discriminants Analysis (QDA)

QDA is a member of discriminants classifiers. It is similar to linear discriminant analysis (LDA). LDA uses a linear hyper-plane to distinct the data into two or more classes. LDA uses homogeneous variance-covariance matrices [49]. However, QDA uses nonlinear hyper-planes such as the ellipse, parabola, and hyperbola to distinct the data, which are non-linearly separable into two or more classes [50]. It uses heterogeneous variance-covariance as well as LDA. In QDA, a different covariance matrix, $\Sigma_{k}$, is generated for each class label *C* of the data, assuming that the data has Gaussian distribution. The Classification rule for QDA is shown in Equation (19),

(19) $$C\left(x\right)=\arg\;\max_{x}\delta_{x}\left(x\right),$$

where $\delta_{x}\left(x\right)$ is the quadratic discriminant function, which is defined in Equation (20).

(20) $$\delta_{x}\left(x\right)=-\frac{1}{2}\log\left|\Sigma_{k}\right|-\frac{1}{2}\left(x-\mu_{k}\right)\Sigma_{k}^{-1}\left(x-\mu_{k}\right)+\log\pi_{k},$$

The class *C* that maximizes the quadratic discriminant function $\delta_{x}\left(x\right)$ needs to be found. It can be noted from the previous equations that the covariance matrix $\Sigma_{k}$ is not identical, so we cannot get rid of the quadratic terms of the quadratic function.

In this study, diagonal quadratic discriminate analysis (DQDA) was used. DQDA is an extension to the QDA method. In DQDA the diagonal covariance matrices are calculated. All off-diagonal elements of each covariance matrix are set to be zero [51,52]. DQDA usually performs better than QDA in high-dimensional data. The distributions of each class, in this paper, were assumed to be multivariate normal.

##### Random Forest Classifier

The random forest (RF) classifier is a popular classifier that is used extensively in medical applications due to its capability to capture relations between data attributes. Capturing these relations helps clinicians understand how the classification process is done. It also makes them understand the association between features in data [53,54]. The RF classifier is used to predict the class value by building several trees from a given input data feature using the divide-and-conquer approach (DAC). The DAC approach depends on a recursive partitioning of the data input space into many subspaces based on the value of the candidate input data feature. The candidate data feature is chosen by applying goodness metric to the data attribute. Different goodness metrics can be used for ranking, including a likelihood ratio and a gain ratio. The leaves of the constructed trees are labeled either as a class label or a probability distribution of the class.

##### Naïve Bayes

Naïve Bayes (NB) is a probabilistic algorithm used for the classification of data. The naïve Bayes classifier is easy to implement and usually yields good results. This classifier is denoted as the maximum posterior (MAP) decision rule [55]. An NB classifier depends on Bayesian theorem to find a particular event and the unconditional probability of an event in each class. In the Bayes theorem, the conditional probability that an event *Y* belongs to the variable $\;X_{n}$ can be computed by using the following Equation (21).

(21) $$P\left(Y|X_{n}\right)=\frac{P\left(X_{n}|Y\right)P\left(Y\right)}{P\left(X_{n}\right)}\;,$$

where *Y* is a variable indicating the class label, $X_{n}$ is a dependent feature vector with size *n*, and $P\left(Y|X_{n}\right)$ is the probability that the input data *Y* belongs to $X_{n}$.

In order to estimate $P\left(Y|X_{n}\right)$ of an instance in a dataset using Equation (22), the classifier assumes that features are statistically independent of any other feature available in a dataset, given the class variable.

(22) $$P\left(Y|X_{n}\right)=\prod_{i=0}^{k}P\left(Y|X_{n}\right).\;$$

##### Radial Basis Function (RBF) Network

An RBF network is a type of artificial neural network (ANN) which uses a radial basis function as an activation function [56]. An ANN is a network with a number of neurons connected together like the brain. It consists of three layers, as follows: Input, hidden, and output layers. The numbers of input and output neurons are equivalent to the number of features and the number of classes in a dataset. The hidden layer of the network is a non-linear RBF activation function. The training of an ANN is made by altering the weights of the connected neurons using a learning algorithm. The output of the network is a linear combination of the neuron outputs of the hidden layer. The testing process is then preformed to examine the performance of this ANN model for predicting the class of unseen examples.

##### Ensemble Classifiers

Ensemble classifiers methods are useful as they can enhance the classification accuracy of a singular classifier by combining multiple classification models [57]. The ensembles assign a class value by fusing several predictions of all the models of the ensembles. Bagging is an example of these ensemble classifiers. It is based on bootstrap aggregation re-sampling method [56]. Bootstrap aggregation is a method used to generate multiple classification models from different random samples of the data. These constructed models are then used to get an aggregated prediction. Bagging ensembles are constructed using random forest, naïve Bayes, and RBF network classifiers. These ensembles contain 60 learners to enhance and improve the accuracy for the classification process.

Adaboosting is another type of ensemble classification. It is like bagging in which several classification models are constructed with different training sets. However, boosting classifiers are connected together in a cascaded manner. The classified examples of the first classifier in the boosting ensemble are re-sampled using a weighting re-sampling technique where greater weights are given to mis-classified instances of the first classifier, and vice versa. The second classifier in the ensemble receives these re-sampled instances and produces output predictions. Again, the same re-sampling procedure is done to these output predictions. This procedure is repeated until all classifiers in the ensemble are processed.

In order to evaluate the performance of the proposed method, several metrics, such as the classification accuracy (ACC), area under the receiver operating curve (AUC), precision, and sensitivity, are used. The results were validated using a 5-fold cross-validation method that was repeated 10 times. The average of the classification accuracy, precision, sensitivity, and area under the curve are calculated as follows.

**Classification Accuracy** is calculated by dividing the number of instances that are correctly classified over the total number of instances in a dataset. This is done using Equation (23).

(23) $$Accuracy=\frac{TP+TN}{TP+TN+FP+FN},$$

where *TP* is the true positive, which indicates the number of positive targets correctly predicted. *TN* is the true negative, which indicates the number of negative targets correctly predicted. Whereas, *FP* is the false positive, which specifies the number of negative targets incorrectly predicted as positive targets. *FN* is the false negative, which specifies the number of positive targets incorrectly predicted as negative targets.

**Precision (P)** is the number of correctly predicted positive instances over the total number of predicted positive instances. The precision is calculated using the following Equation (24):

(24) $$Precision=\frac{TP}{TP+FP},$$

**Sensitivity (S):** Which is also called recall or true positive rate (TPR). It is the number of positive class instances that are correctly classified over the total number of positive class instances available in the data. It is calculated using Equation (25).

(25) $$Sensitivity=\frac{TP}{TP+FN}.$$

**Area Under the Receiver Operating Characteristic Curve (AUC)**: A receiver operating characteristic (ROC) curve is a graph representing the TPR as a function of the false positive rate (FPR). AUC is the measure of the difference between the distributions of the two classes for classification [58]. In other words, it measures the area located below the ROC curve.

## 3. Results

In this article, 227 (113 healthy and 114 unhealthy) fetal MRI scans the trials were conducted with various GA and positions. The performance of the classification stage depended on the segmentation stage. For this reason, the performance of the segmentation stage for the proposed method was measured using the Dice metric (D) and the segmentation accuracy (SA) were calculated between the ground truth. The results of the segmentation method are shown in Table 1. It was observed that, out of 50 MRI images, the mean values for Dice (D) and accuracy (AS) were 0.975 and 0.989, respectively. Additionally, the Jaccard Index (JAC), precision (PPV), sensitivity (S), and specificity (Sp) values were 0.951, 0.994, 0.972, and 0.979, correspondingly. These values are close to 1, which shows that our proposed method can successfully segment fetal brain images.

The performance of the new presented approach was calculated using the classification accuracy (ACC) for the five individual classification models for the six subsets of features, as shown in Table 2. These individual classifiers include DQDA, naïve Bayes, KNN, random forest, and RBF network classifiers. As mentioned before, feature subsets two and six had high dimensional space, therefore the PCA feature reduction method was used to reduce their dimension. It is clear from the table that feature subset six has a higher ACC (91%, 95.6%, 93%, 91.2%, and 90.3% using QDQA, KNN, RBF network, naïve Bayes, and random forest) compared to the other subsets of features. Feature subset six represents features extracted from the Gabor filter feature extraction method, combined with the GLCM and statistical descriptive features extracted from DWT coefficients (fifth subset). The results showed that combining several textural features is better than using one type of feature extraction method. The highest classification accuracy, 95.6%, was achieved using the KNN classifier.

The sensitivity (*S*), precision (*P*), area under ROC (*AUC*) were also calculated for the feature subset six (the results are shown in Table 3). This features set has achieved the highest accuracy compared to other feature subsets (see Table 1). The results of Table 3 show that the classifiers have an acceptable performance in classifying the abnormalities. The *AUC* of KNN was 99%, which was greater than the other classifiers (98.5%, 93%, 97.6%, and 96.3% of the DQDA, RBF network, naïve Bayes, and random forest classifiers, respectively). Additionally, the DQDA classifiers had a *P* of 99%, which was higher than the other classifiers (97%, 94%, 91.2%, and 91% of the K-NN, RBF network, naïve Bayes, and random forest classifiers, respectively). Finally, the *S* of the DQDA, K-NN, RBF network, naïve Bayes, and random forest classifiers were 83%, 94%, 93%, 92%, and 90.3%, respectively.

Ensemble classifiers combine the power of individual classification models available in the ensemble. This prevents the possibility of poor results that could be produced from a certain unsuitably individual model. Therefore, bagging and Adaboosting ensembles were constructed using naïve Bayes, random forest, and RBF networks. Their performances were compared with each other and the individual version of these classifiers. These results are presented in Table 4. Table 4 shows that the performance of bagging and Adaboosting ensembles constructed using naïve Bayes, RBF neural network, and random forest classifiers outperformed the performance of their individual models. This is because the ACC of the bagged and Adaboosted naïve Bayes were 92.1% and 92.5%, which are greater than the 91.63% of the individual naïve Bayes. In addition, the ACC of the bagged and Adaboosted random forest were 92.5% and 91.2%, which are greater than the 90.3% of the individual random forest classifier. In addition, the ACC of the Adaboosted RBF network is 93.4% which is greater than that of the individual RBF network classifier. However, the ACC of the bagged, RBF network was 93%, which was the same as the individual RBF Network classifier. The highest *P*, of 93.6%, was achieved by the RBF network, the bagged RBF network, and bagged naïve Bayes classifiers. The highest *S* of 93.4% was achieved by the Adaboosted RBF network.

To test and validate the statistical significance of the results, a one-way analysis of variance test was performed on the results obtained from the repeated fivefold cross-validation process. The null hypothesis *H*_o_ for this experiment was that the mean accuracies of all the classifiers were the same. This test was first performed on the accuracy results of individual classifiers to test the statistical significance between them. The results are shown in Table 5. Then the test was done on the results of the ensemble classifiers to test the statistical significance between them. The results of the test are shown in Table 6. Finally, the test was applied on the results of both individual classifiers and their ensemble. The results of the test are shown in Table 7. It can be observed from Table 5, Table 6 and Table 7 that the *p*-values achieved were lower than α, where α = 0.05. Therefore, it can be concluded that there was a statistically significant difference between the accuracies of the classifiers.

As it is commonly known, in order to construct an efficient classification model that achieves high classification accuracy, the number of images for each class label used to construct the model should be large enough. However, the dataset used in the proposed approach contained several types of abnormalities with a limited number of images for each abnormality. Therefore, we gathered all images of several abnormalities, of not only one type, along with normal images, and classified them to either normal or abnormal brains. Note that the datasets that are freely available online contain images of normal and not abnormal, which is why were constrained to classify between normal and abnormal only.

The main aim of this article is to classify fetal brain abnormalities. Several constraints arose for reaching this aim. First, we were restricted with the limited number of related research articles discussing this topic (3 articles, as stated in the introduction). The majority of the previous research has been limited to studies classifying brain deficiencies in early age for the preterm and neonatal stages, but not at the fetus stage. The second constraint was that datasets that contain images of fetal brains, as well as those from neonates and preterm infants are not available online. Finally, datasets that are available online for the fetal stage contain only images of normal brains, so we were not able to use them as our main target was to classify fetal brain images. Therefore, we compared our results with the performance of such approaches for fetal, neonates, and preterm infants to validate and indicate that the performance of our method is competitive. The results are displayed in Table 8.

The proposed method has an *ACC* that ranges between 90.3% and 95.6%, which is higher than the *ACC* of the previous methods proposed for preterm and newborn infants [12,13,14,15] and which achieved 80%, 84%, 71%, and 76%, respectively. The AUC of the proposed method is also higher than the 80% achieved by the method used for classifying fetal abnormalities [10]. The new approach presented in this article identified different brain deficiencies, unlike [12,13,14,15] methods that were limited to the classification of only one type of defect for preterm infants or newborns. Lastly, our new approach employed a larger number of MRI images compared to other approaches. Identifying fetal brain defects is a complicated task compared to scanning of newborns and preterm infants. Since scanning of fetal brains displays the head of the fetus along with the maternal parts of the mother and other surrounding fluids [7], our approach, therefore, has several strengths over other methods reported in the previous related work. First, it is considered to be simple and reliable. Second, the results showed that it works well on T2 weighted scans. Third, it is capable of classifying different GA and several brain deficiencies. Finally, our approach classifies the defect before the fetus is born.

## 4. Discussion

The proposed framework is for the classification FBA. This framework uses several types of machine learning techniques. Most of the work done for brain MRI detection and classification in a primary age is for newborns and preterm infants. To the best of our own knowledge, the work done for FBC is limited to References [9,10,11]. In the articles [9,10], the authors identified a brain deficiency called SGA in neonates, as opposed to fetuses. Their approach was applied on a very small number of images. These images were only limited to 37 weeks of fetal GA. Reference [11] classified several fetal abnormalities, but the highest classification accuracy achieved was limited to 80%, which is insufficient for medical applications. Therefore, in this article, a new approach was proposed to classify brain defects from fetal images at various GAs.

The proposed framework consists of five steps, as follows: Segmentation, enhancing the minor ROI image contrast, feature extraction, feature reduction, and classification. In the segmentation phase, the minor ROI was cropped semi-automatically from the remaining parts of the fetal images. Afterwards, the minor ROI images were improved by a mixture of contrast stretching methods. Next, in the feature extraction phase, six subsets of features were extracted. The first subset represents the horizontal, vertical, approximate, and diagonal coefficients of the 4th decomposing stage of the DWT. The second subset feature was extracted using Gabor filters. The third subset of features was extracted using the gray-level co-occurrence matrix (GLCM) and statistical features. The forth subset is a combination of features extracted from DWT, GLCM, and statistical features. The fifth subset is the GLCM and statistical features extracted from the fourth level 2D-DWT decomposition of the minor ROI. The sixth subset represents features extracted using a Gabor filter combined with the GLCM and statistical descriptive features extracted from the DWT coefficients (fifth subset); that is to say that the sixth subset is a combination of the Gabor filter feature vector with the fifth subset. Note that feature subsets two and six are of high dimension; therefore, principal component analysis (PCA) was applied to them to reduce their dimension. Finally, the six subsets of features were used to train and validate different classification models, such as naïve Bayes, random forest decision tree, RBF network, DQDA, and (K-NN) classifiers. In addition, bagging and Adaboosting ensembles were constructed using naïve Bayes, random forest decision tree, and RBF network classifiers to see if they could enhance predictions of their individual models. The proposed pipeline reached a classification accuracy of 95.6%, which outperformed other methods shown in the literature. Therefore, it can be considered as a powerful tool that can help physicians in the early detection and diagnosis of fetal brain abnormalities.

Proper comparison with other previous work is mandatory and this comparison is more appropriate when it is based on the same dataset. However, as we explained before, our key goal was to classify fetal brain abnormalities. We faced a problem when searching for related research papers discussing this topic. We found that there is a limited number of papers which have explored fetal brain classification (only three relevant papers, as mentioned before). The authors of these papers used a machine learning classifier for either predicting one type of abnormality after the birth of the infant (becoming a neonate), which is not similar to our case (we want to differentiate between several types of abnormalities and normal brains and we strive to classify fetal abnormality before the fetus is born), or to classify several abnormalities and achieve a classification accuracy limited to 80% (which is not very high, especially in medical applications). The datasets are not freely available online. Moreover, all the fetal brain datasets that are available online only include images of normal brains. The dataset used in our work is the only dataset that is free online and contains abnormal images. In addition, the literature showed that the majority of work done on detecting and classifying brain abnormalities in early age was limited to infants, neonates, and preterm infants, rather than fetuses. We thought that the previous work done for infants, neonates, and preterm infants would be the closest work we could use for comparison. Therefore, we compared these approaches (examples [12,13,14,15]), but their data was not freely available online. We also compared with other papers that used fetal images (examples [9,11]).

As it is clear from the comparison with recent related work, most of them used the SVM classifier to construct their models. To make our study novel, we did not employ SVM classifier. SVM is a popular classifier; however, it would not be highly efficient if the number of features is large [59]. In our case, several features sets were extracted. Most of these features had a high number of features. Moreover, SVM would produce insufficient results when the datasets are partitioned into subsets and used in parallel training, where each subset is used to train a classification model and then the knowledge is combined together [60]. In our case, the dataset was split using a bootstrap aggregating resampling technique. These subsets were then used to train the bagging ensemble models. In addition, it is difficult to choose the parameters as well as the best kernel of SVM [61]. For these reasons, SVM was not adopted in our article.

MATLAB R2017b and Waikato Environment for Knowledge Analysis (Weka) software [62] were used for the implementation of the proposed framework.

## 5. Conclusions

This paper presents a framework for identifying defects in fetal brains. The aim of this work is to classify these defects in a primary stage, before the fetus fetal is born, via a simple and fast approach. The results of the new approach presented in this article indicate a good performance using MRI images. The proposed framework has effectively identified defects of fetal brains within MRIs of diverse planes and ages (from 16 weeks to 39 weeks). In addition, it had the ability to detect and identify numerous defects in the fetal brain, as opposed to only one type of abnormality. In addition, the ensemble classifiers constructed in the proposed algorithm proved to beat the classification results of their individual models in most cases. To the best of our knowledge, most of the previous work focused on classification of preterm and neonatal MRI brain images. The few studies that investigated fetal MRI scans for identification process associated fetal scans with their newborn scans to estimate the deficiency, called SGA, in newborns as opposed to fetuses. For this reason, our approach performance was measured against other recent related approaches for preterm babies and newborns. Our classification performances indicated that our new approach outperformed all them. This work will pave the way for future studies, encouraging academics to begin working on classifying the fetal brain to detect defects. In addition, it will help doctors make an accurate diagnosis, decide the appropriate treatment plan, and properly advise parents on how to deal with the abnormality before the fetus is born. Future work will focus on enhancing prediction accuracy and applying deep learning methods for fetal brain classification.

## Figures and Tables

**Table 1:** Results of the Dice (D), Jaccard Index (JAC), precision (PPV), sensitivity (S), specificity (Sp) and accuracy segmentation (AS), along with the standard deviation (std), for the segmentation method for 50 MRI images.

Image Label	D (std)	JAC (std)	Sp (std)	S (std)	AS (std)	PPV (std)
**Mean (Std.)**	0.975(0.02)	0.951(0.03)	0.994(0.01)	0.972(0.03)	0.989(0.01)	0.979(0.02)

**Table 2:** Classification accuracy of individual classifiers constructed using different combinations of feature extraction methods.

Classifier Model	Feature Subset 1 (DWT Features	Feature Subset 2 (Gabor Features + PCA)	Feature Subset 3 (GLCM + Statistical Features)	Feature Subset 4 (DWT + GLCM + Statistical Features)	Feature Subset 5 (GLCM and Statistical Features Extracted from DWT)	Feature Subset 6 (Gabor Features + Feature Subset 5+ PCA)
**QDQA**	71%	89%	50%	50%	74%	91%
**K-NN**	62%	94.3%	63%	63%	65%	95.6%
**RBF Network**	70%	92%	67%	71%	71%	93%
**Naïve Bayes**	71%	90%	63%	72%	73%	91.2%
**Random Forest DT**	70%	86.3%	71.8%	74%	72.2%	90.31%

**Table 3:** Metrics for feature subset 6, which has the highest classification accuracy.

Classifier Model	ACC (std)	P (std)	S (std)	AUC (std)
**QDQA**	91%(0.0092)	99%(0.0052)	83%(0.00516)	98.5%(0.0023)
**K-NN**	95.6%(0.0056)	97%(0.0067)	94%(0.024585)	99%(0.0067)
**RBF Network**	93%(0.006)	93.6%(0.005587)	93%(0.0066)	93.3%(0.0082)
**Naïve Bayes**	91.2%(0.0104)	92.2%(0.008072)	91.6%(0.0098)	97.2%(0.0077)
**Random Forest DT**	90.31%(0.001)	91%(0.008634)	90.3%(0.0086)	96.3%(0.0099)

**Table 4:** Comparison of the classification results along with the standard deviation (std) of the individual classifiers and their ensembles.

Classifier Model	ACC (std)	P (std)	S (std)
**RBF Network**	93%(0.006)	93.6%(0.005587)	93%(0.00594)
**Bagged RBF Network**	93%(0.0066)	93.6%(0.00607)	93%(0.006596)
**Adaboosted RBF Network**	93.4%(0.0045)	93.6%(0.006346)	93.4%(0.00674)
**Naïve Bayes**	91.63%(0.0104)	92.2%(0.008072)	91.6%(0.009776)
**Bagged Naïve Bayes**	92.1%(0.008)	92.7%(0.004673))	92.1%(0.006015)
**Adaboosted Naïve Bayes**	92.5%(0.0084)	93%(0.006033)	92.5%(0.00819)
**Random Forest DT**	90.31%(0.001)	91%(0.008634)	90.3%(0.008634)
**Bagged Random Forest DT**	92.5%(0.0058)	92.6%(0.006129)	92.5%(0.005181)
**Adaboosted Random Forest**	91.2%(0.0077)	91.3%(0.008)	91.2%(0.0075)

**Table 5:** One-way analysis of variance test details for the individual classifiers.

Source of Variation	SS	df	MS	F	*p* Value
Columns	0.01799	10	0.0018	34.29	<0.001
Error	0.00311	99	0.00005		
Total	0.02318	109			

**Table 6:** One-way analysis of variance test details for the ensemble classifiers.

Source of Variation	SS	df	MS	F	*p* Value
Columns	0.00516	8	0.00065	12.26	<0.001
Error	0.00426	81	0.00005		
Total	0.00943	89			

**Table 7:** One-way analysis of variance test details for the individual classifiers and their ensembles.

Source of Variation	SS	df	MS	F	*p* Value
Columns	0.001561	4	0.0039	56.5	<0.001
Error	0.00311	45	0.00007		
Total	0.01872	49			

SS sum of squares, df degree of freedom, MS mean squared error, F F-statistic.

**Table 8:** Comparisons between the performances of our novel presented approach and recent related approaches for preterm, neonates, and fetal.

Paper	Abnormality Type	GA	Classifier	Accuracy (ACC)
12	Functional connectivity in preterm infants	preterm of age 30 to 39 weeks	Non-linear SVM	80%
13	Brain Maturity	preterm of age 23–29 weeks	Linear SVM	84%
14	Autism spectrum disorder	23–31 weeks preterm	Linear SVM	71%
15	Autism spectrum disorder	6-month-old infants	Multi-kernel SVM	76%
9	Small of gestational age	37 weeks	Support Vector Machines (SVM) & Genetic Algorithms (GALs)	95.12% for frontal lobe, 95.56% for basal ganglia, 93.18% for mesencephalon and 83.33% for cerebellum
11	Several Fetal Abnormalities	16–39 weeks	LDA	79%
			Linear SVM	79%
			KNN	73%
			Ensemble Subspace Discriminates	80%
**Our method**	Several Fetal Abnormalities	16–39 weeks	DQDA	92%
			K-NN	95.6%
			RBF Network	93%
			Naïve Bayes	91.63%
			Random Forest DT	90.3%
			Bagged RBF Network	93%
			Adaboosted RBF Network	93.4%
			Random Subspace RBF	93%
			Bagged Naïve Bayes	92.1%
			Adaboosted Naïve Bayes	92.5%
			RS Naïve Bayes	91.2%
			Bagged Random Forest	92.5%
			Adaboosted Random Forest	91.2%
			Random Subspace RF	93.4%
